# Depression and Peripheral Artery Disease: A Narrative Review

**DOI:** 10.7759/cureus.93652

**Published:** 2025-10-01

**Authors:** Alexandros Giosdekos, Christos Bakoyiannis, Emmanouil Rizos, Ioannis D Kakisis

**Affiliations:** 1 2nd Department of Vascular Surgery, Attikon University Hospital, Athens, GRC; 2 1st Department of Surgery, National and Kapodistrian University of Athens, Laikon General Hospital, Athens, GRC; 3 Department of Psychiatry, Attikon University Hospital, Athens, GRC

**Keywords:** depression, peripheral artery disease, restenosis, revascularization, review article

## Abstract

Peripheral artery disease is a chronic vascular disorder associated with substantial morbidity, mortality, and functional decline. Depression frequently coexists with this condition and is consistently linked to adverse outcomes, including increased risk of limb loss, higher mortality, reduced adherence to therapy, and diminished benefits from revascularization procedures. This narrative review summarizes the current evidence on the prevalence of depression among patients with peripheral artery disease, its impact on clinical outcomes, and the biological and behavioral mechanisms that may explain this association. The findings emphasize the importance of recognizing depression as a major determinant of prognosis in peripheral artery disease and support systematic screening and integrated management as essential components of comprehensive patient care.

## Introduction and background

Peripheral artery disease (PAD) is a prevalent and serious vascular condition, affecting over 200 million people worldwide. In the United States, an estimated 7 to 12 million individuals are affected, while in Europe, approximately 40 million people live with PAD (~5.3% of the population, including 17 million within the European Union). The prevalence varies widely across European countries, ranging from 7.0% in Belgium and 8.1% in the Netherlands to 22.9% in Italy and as high as 28% in Greece. In the United Kingdom, about 20% of individuals over 60 years have PAD, while in Germany, prevalence increases from ~3% in mid-life to >18% among men aged 70-75 years. Such figures highlight the substantial and heterogeneous burden of PAD across regions [1-3]. In advanced stages, PAD can lead to severe complications, including ischemic ulcers, gangrene, tissue loss, and limb amputation [1,2,4]. Despite advances in revascularization techniques and pharmacological therapies, the disease’s impact on patient-centered outcomes, such as functional status, independence, and mental health, remains underexplored compared with other cardiovascular conditions [5,6].

Recent evidence suggests that depression, a condition affecting approximately 8.4% of the U.S. adult population, is disproportionately prevalent among individuals with PAD [7]. Studies have reported depression rates in PAD patients ranging from 3% in general population-based samples to as high as 48% in patients with advanced disease [8-11]. More consistent estimates from well-characterized PAD cohorts fall between 20% and 37% [8,9], and two recent meta-analyses found a pooled prevalence of ~24-25% [12-14]. This comorbidity is associated with poorer functional status and worse clinical outcomes, including heightened risks of major adverse cardiovascular events (MACE), amputation, and mortality [11,15]. Furthermore, PAD patients with depression often experience challenges adhering to treatment regimens, including exercise therapy, smoking cessation, and lifestyle modification, which may accelerate disease progression [4,5].

**Figure 1:**
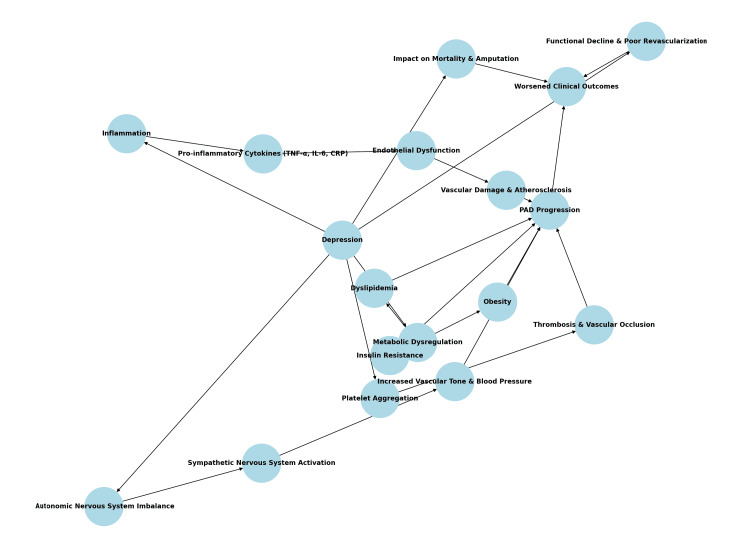
Pathogenetic mechanisms and clinical impact of depression on peripheral artery disease (PAD) Original schematic illustration created by the authors using Microsoft Paint (Microsoft Corp., Redmond, WA, USA)

The interplay between PAD and depression underscores the critical need for comprehensive management strategies that address both physical and psychological health. While the association between depression and adverse outcomes is well documented in other cardiovascular diseases, such as coronary artery disease and heart failure, its implications in PAD have been less thoroughly investigated [6,16]. Understanding the prevalence and impact of depression in PAD is essential for optimizing care and improving patient outcomes. This review aims to synthesize available evidence on the prevalence of depression in PAD, assess its impact on clinical outcomes such as mortality, amputation, and functional decline, and explore the underlying biological and behavioral mechanisms that may drive these associations (Figure 1).

## Review

Methods

This narrative review aimed to summarize the prevalence of depression in patients with peripheral artery disease (PAD), evaluate its impact on major clinical outcomes such as mortality, amputation, and functional decline, and describe the underlying biological and behavioral mechanisms linking depression to PAD. A narrative review design was chosen because the available evidence is heterogeneous, spanning observational cohorts, systematic reviews, meta-analyses, and Mendelian randomization studies. Such diversity precludes de novo quantitative pooling but allows for a structured thematic synthesis across methodologies.

**Figure 2:**
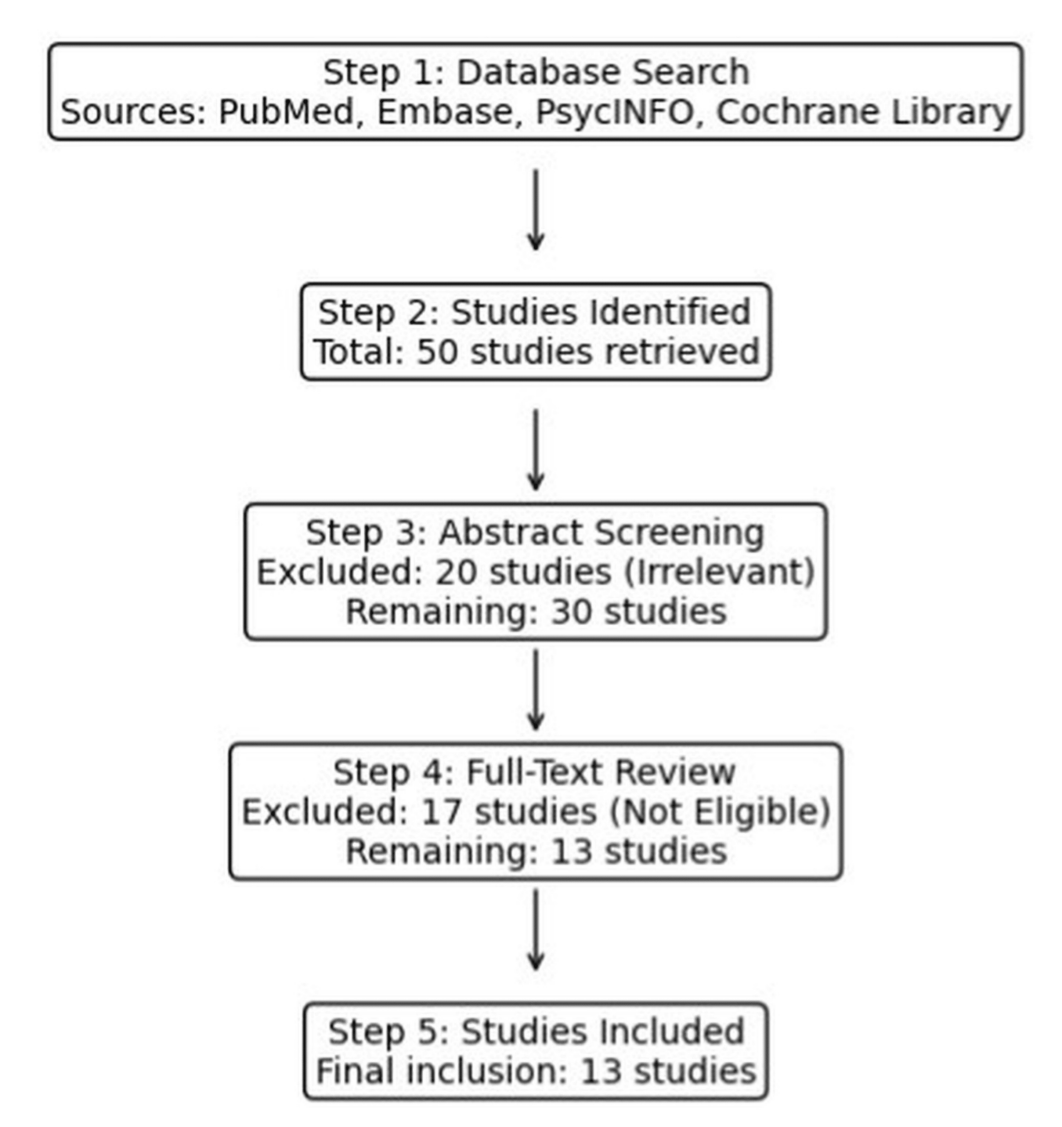
Flow diagram of study selection

The review was conducted in accordance with the SANRA (Scale for the Assessment of Narrative Review Articles) guidelines, ensuring methodological quality through a focused research question, transparent eligibility criteria, systematic database searches, and appropriate referencing [17]. Eligible studies included adult patients with a confirmed diagnosis of PAD, with or without surgical or endovascular revascularization, who reported on depression prevalence, pathophysiological mechanisms, or clinical outcomes. Only studies using validated depression measurement tools (e.g., Patient Health Questionnaire-9 or PHQ-9, Geriatric Depression Scale-Short Form or GDS-SF, Center for Epidemiologic Studies Depression Scale or CES-D, or International Classification of Diseases-coded diagnoses) were included. Case reports, conference abstracts, and studies without explicit data on both PAD and depression were excluded, as were studies focused exclusively on psychological conditions other than depression. A comprehensive search was performed in PubMed/MEDLINE, Embase, PsycINFO, and the Cochrane Library for articles published between January 2000 and January 2024. This timeframe was chosen to reflect contemporary PAD management and the increasing recognition of psychological determinants of vascular outcomes. The search strategy combined controlled vocabulary (MeSH in PubMed, Emtree in Embase) with free-text terms. Three concept blocks were used: disease terms (“Peripheral Artery Disease,” “PAD”), exposure terms (“Depression,” “Major Depressive Disorder,” “MDD,” “Mental Health”), and outcome terms (“Mortality,” “Amputation,” “Functional Outcomes”). Boolean operators (AND/OR) were applied to combine these categories, and equivalent subject headings were used across databases. Reference lists of included studies and relevant reviews were screened manually to identify additional eligible articles (Figure 2).

Data were extracted systematically from each study, including study design, year of publication, sample size, population characteristics, depression measurement tools, key outcomes, and effect estimates (e.g., hazard ratios, odds ratios). Thematic synthesis was used to organize the findings into distinct categories: prevalence of depression in PAD, biological and behavioral mechanisms linking depression to PAD, impact of depression on clinical outcomes, mediating risk factors such as smoking and body mass index, and implications of depression screening and management for PAD care.

This review synthesized evidence from 13 primary studies that met the inclusion criteria. Pooled effect estimates presented in the Results are derived from published meta-analyses [12-14] rather than from a new quantitative analysis conducted by the authors (Table 1). Given this heterogeneity, pooled estimates were not calculated.

**Table 1:** Study design and sample size summary QoL: quality of life; PAD: peripheral artery disease; MALE: major adverse limb events; MDD: major depressive disorder; NL: Netherlands

Authors	Year	Country	Study design	Sample size	Notes/focus
McDermott et al. [18]	2002	USA	Cross-sectional	423	Depression and functional decline
Cherr et al. [4]	2007	USA	Retrospective cohort	216	Revascularization outcomes, QoL
Ruo et al. [19]	2007	USA	Prospective cohort	417	Depression and functional decline
Smolderen et al. [8]	2008	Netherlands	Prospective cohort	166	Prevalence and underdiagnosis of depression
Smolderen et al. [20]	2011	Netherlands	Prospective cohort	242	Depression impact on health status, QoL
McDermott et al. [9]	2016	USA	Prospective cohort	1,429	Functional decline, walking distance
Arya et al. [15]	2018	USA	Retrospective cohort (Veterans)	155,647	Mortality, amputation
Abi-Jaoudé et al. [12]	2022	International	Systematic review/meta-analysis	~11 studies (~10,000 PAD patients)	Prevalence and MALE risk
Welch et al. [11]	2023	USA	Cross-sectional	104	Prevalence, QoL
Scierka et al. [13]	2023	USA, NL	Systematic review/meta-analysis	119,123 patients	Mortality and prevalence
Chyrek-Tomaszewska et al. [10]	2024	Poland	Cross-sectional	159	Prevalence (European cohort)
Shakt et al. [14]	2024	USA	Mendelian randomization	135,458 (MDD), 31,307 (PAD)	Genetic links, mediation via smoking/BMI

Results

This review synthesized findings from 13 primary studies examining the relationship between depression and PAD, along with evidence from three published meta-analyses [12-14]. The results are presented according to the review aims, focusing on prevalence, clinical outcomes (mortality, amputation, functional decline), and underlying mechanisms. Depression measurement tools and study characteristics are summarized in Table 2, while prevalence data are highlighted in Table 3.

**Table 2:** Depression measurement tool and key findings summary PAD: peripheral artery disease; QoL: quality of life; HR: hazard ratio; ICD codes: International Classification of Diseases codes; MALE: major adverse limb events

Study	Population/setting	Depression assessment tool	Key outcome(s)	Effect size (if reported)	Notes
McDermott et al. (2002) [18]	PAD patients, USA	Validated questionnaire	Functional decline	—	Early evidence of depression impact on function
Cherr et al. (2007) [4]	Revascularization cohort, USA	Validated questionnaire	QoL, recurrent symptoms	37% depressed	Depression linked to poor QoL post-surgery
Ruo et al. (2007) [19]	Prospective PAD cohort, USA	Validated questionnaire	Functional decline	—	Persistent depression predicted decline
Smolderen et al. (2008) [8]	PAD cohort, Netherlands	Validated questionnaire	Prevalence, underdiagnosis	28.4%	Underdiagnosis more common in men
Smolderen et al. (2011) [20]	PAD cohort, Netherlands	Validated questionnaire	Health status, QoL	—	Depression reduced QoL
McDermott et al. (2016) [9]	Large PAD cohort, USA	Validated questionnaire	Functional decline	HR 1.5	Slower walking, poorer recovery
Arya et al. (2018) [15]	US Veterans, USA	ICD codes	Mortality, amputation	HR 1.7 (mortality), HR 2.0 (amputation)	Strongest evidence from large cohort
Abi-Jaoudé et al. (2022) [12]	International meta-analysis (~11 studies)	Mixed tools	Prevalence, MALE risk	≈25% pooled	Higher risk of MALE events
Welch et al. (2023) [11]	Cross-sectional PAD cohort, USA	Validated questionnaire	QoL, prevalence	—	Contemporary prevalence estimate
Scierka et al. (2023) [13]	Systematic review/meta-analysis (~119,000 pts)	Mixed	Mortality, prevalence	HR 1.24 (mortality)	Synthesized large population data
Chyrek-Tomaszewska et al. (2024) [10]	PAD cohort, Poland	Validated questionnaire	Prevalence	—	Small European study
Shakt et al. (2024) [14]	Genetic data, USA	Mendelian randomization	PAD risk	—	Depression → PAD via smoking, BMI

**Table 3:** Prevalence of depression in patients with PAD across included studies PAD: peripheral artery disease; QoL: quality of life; MALE: major adverse limb events

Study	Design/population	Depression assessment	Reported prevalence	Key notes
Smolderen et al. (2008) [8]	Cross-sectional PAD cohort	Validated questionnaire	28.4%	Underdiagnosis more common in men
Cherr et al. (2007) [4]	Revascularization cohort	Validated questionnaire	37%	Linked to worse QoL and recurrent symptoms
McDermott et al. (2016) [9]	Prospective PAD cohort	Validated questionnaire	~20–30%	Associated with functional decline
Abi-Jaoudé et al. (2022) [12]	Systematic review/meta-analysis (~11 studies)	Mixed tools	≈25% pooled	Higher risk of MALE events
Scierka et al. (2023) [13]	Systematic review/meta-analysis (~119,000 pts)	Mixed tools	~25% pooled	Nearly doubled prevalence vs. non-PAD
Shakt et al. (2024) [14]	Meta-analysis (~10,000 PAD patients)	Mixed tools	24% pooled	Depression associated with poor outcomes

Prevalence of Depression in PAD

Depression was prevalent among patients with PAD, with rates ranging from 20% to 37% depending on the population and the measurement tools used [8,9]. In patients undergoing lower extremity revascularization, 37% demonstrated significant depressive symptoms [4]. Another cross-sectional study reported a prevalence of 28.4%, with underdiagnosis particularly common among male patients [8].

Meta-analyses confirmed these findings. Abi-Jaoudé et al. reported a pooled prevalence of ~25% across 11 studies, while Shakt et al. found a prevalence of 24% in >10,000 PAD patients [12,14]. Scierka et al. similarly reported ~25% pooled prevalence and nearly doubled depression risk in PAD compared with non-PAD populations [13]. These results confirm depression as a frequent comorbidity (Table 3).

Impact of Depression on Mortality and Amputation

Depression significantly increased the risk of mortality and major amputation in PAD. In a large U.S. Veterans cohort (>150,000 patients), depressed individuals had a 70% higher risk of all-cause mortality (HR 1.7, 95% CI 1.5-2.0) and a two-fold increased risk of major amputation (HR 2.0, 95% CI 1.8-2.3) compared to non-depressed peers [12-14]. Findings from several meta-analyses reinforced these associations, showing elevated risks of both mortality and major adverse limb events (MALE), such as major or minor amputation, any amputation, or amputation plus repeat peripheral vascular intervention.

Across pooled data, depression was associated with a 24% increase in all-cause mortality (HR 1.24, 95% CI 1.07-1.25) and up to a 50% increased risk of MALE, although one review noted that the link with limb events did not consistently reach statistical significance [12-14].

Underlying mechanisms are multifactorial. Behavioral pathways include poor adherence to medications, reduced participation in exercise therapy, and persistent smoking [4,13]. Biological pathways involve HPA-axis activation, elevated cortisol, autonomic dysregulation, and increased inflammatory cytokines (e.g., TNF-α, IL-6, CRP), which impair endothelial function, collateral formation, and wound healing [16,21]. Genetic evidence also suggests that part of the risk is mediated by smoking and higher BMI, although some independent effect of depression remains [14].

Functional Decline and Revascularization Outcomes

Depression was consistently associated with functional decline in PAD patients. Depressed individuals experienced reduced walking distances and slower velocities; for example, depression was linked to a 40% reduction in pain-free walking distance (p = 0.02) and a 1.5-fold increased risk of functional decline (HR 1.5, 95% CI 1.2-1.9) [9].

Depressive symptoms were also associated with smaller improvements in quality of life and walking distances following revascularization procedures [4,11,20]. This reduced responsiveness underscores the need to integrate mental health management into PAD rehabilitation.

The role of antidepressant therapy remains uncertain. In a post-hoc analysis of the BIP randomized trial, patients with pharmacologically treated depression (14.5% of participants) demonstrated a greater decline in daily step counts over two years compared to non-depressed peers, despite no significant difference in six-minute walk distance [22]. Similarly, the Veterans cohort found that while antidepressant-treated depression was associated with higher risks of mortality and amputation, the effect size was attenuated compared with untreated depression [15]. These findings suggest that while pharmacotherapy may improve mood and adherence, its independent effect on PAD outcomes remains inconclusive [21,22].

Discussion

This review highlights the significant relationship between depression and peripheral artery disease (PAD), emphasizing its profound impact on patient outcomes. Depression is disproportionately prevalent in PAD, contributing to poorer functional and surgical outcomes, higher mortality, and an increased risk of major amputation [4,9,15]. These findings align with previous research indicating that depression exacerbates PAD not only through biological pathways such as inflammation and endothelial dysfunction but also via adverse health behaviors, including poor adherence to therapy, smoking, and physical inactivity [5,21].

Prevalence of Depression in PAD

Across observational studies, depression prevalence ranged from 3% to 48% depending on study setting and methodology [8-10]. More consistent estimates from well-characterized PAD cohorts fall within 20-37% [4,8], and three meta-analyses reported a pooled prevalence of ~24-25% [12-14]. These rates are markedly higher than in the general population and frequently underdiagnosed, particularly in men [8,11]. Routine screening is therefore warranted. Practical approaches include brief validated tools such as the PHQ-2 or PHQ-9, which can be administered during outpatient or pre-revascularization visits, embedded in EMRs, and linked to referral pathways for positive screens.

Mortality and Amputation Outcomes

Depression independently predicted all-cause mortality and major amputation. In a large U.S. Veterans cohort, depressed patients had a 70% higher mortality risk and nearly doubled risk of major amputation [15]. Moreover, a systematic review and meta-analysis including more than 119,000 patients confirmed these findings, reporting a 24% increased mortality risk (HR 1.24, 95% CI 1.07-1.25) but no statistically significant increase in MALE [13]. Similarly, Shakt et al. found a pooled HR of 1.24 for mortality and a 50% increased risk of MALE [14]. Together with Abi-Jaoudé et al., these meta-analyses establish depression as an independent prognostic factor for poor PAD outcomes [12,14].

Functional Decline and Revascularization Outcomes

Depression was strongly associated with functional decline, reduced walking distances, and diminished quality of life [9]. After revascularization, depressed patients reported smaller improvements in walking ability and greater recurrence of symptoms [4,11,20]. This reduced responsiveness to surgical or endovascular treatment underscores the need to integrate mental health management into PAD rehabilitation.

Mechanisms Linking Depression and PAD

Mechanistic pathways are multifactorial. Behavioral factors include persistent smoking, sedentary lifestyle, and non-adherence to medication [4,5]. Biological factors include hypothalamic-pituitary-adrenal (HPA) axis dysregulation, elevated cortisol, autonomic imbalance, and increased proinflammatory cytokines (TNF-α, IL-6, CRP), which impair collateral vessel formation and wound healing [16,21]. Immune dysregulation, rather than isolated complement or fibrosis pathways, appears to be the main inflammatory mechanism. Genetic evidence from Mendelian randomization also suggests shared predisposition, with smoking and BMI mediating part of the effect of depression on PAD, although some independent effect remains [14,23].

Antidepressant Therapy

Whether antidepressant therapy improves PAD outcomes is unclear. Large-scale data suggest that treated depression carries a lower risk than untreated depression, but outcomes remain worse than in non-depressed patients. In the U.S. Veterans cohort, antidepressant use attenuated-but did not eliminate-excess mortality and amputation risk [15]. The BIP trial analysis found that antidepressant-treated depressed patients experienced greater declines in daily step counts than controls, despite similar six-minute walk distances [22]. This suggests pharmacotherapy improves mood but does not fully reverse PAD-related risk. Poor adherence likely diminishes potential benefit. Thus, combined treatment strategies (pharmacotherapy, cognitive-behavioral therapy, exercise, and lifestyle modification) may offer the best outcomes [21,22].

Clinical Implications

Given the high prevalence and adverse prognostic significance of depression, PAD care should incorporate systematic screening, early referral, and multidisciplinary management. Tools such as the PHQ-2 or PHQ-9 can be seamlessly integrated into vascular outpatient visits or preoperative assessments. Addressing depression could improve adherence to medications and rehabilitation, enhance quality of life, and reduce risks of mortality and limb loss.

Limitations and Future Research

This review is limited by the observational design of most included studies, heterogeneity in depression measurement tools, and inconsistent adjustment for confounders. Evidence on antidepressant treatment remains scarce and mixed. Future research should include randomized controlled trials testing integrated depression management in PAD, stratifying outcomes by antidepressant class and adherence, and examining mechanistic biomarkers (inflammation, endothelial function). A concise summary of the key findings and their clinical implications is provided in Table 4.

**Table 4:** Summary of key findings and clinical implications PAD: peripheral artery disease; HPA axis: hypothalamic–pituitary–adrenal axis; CBT: cognitive behavioral therapy

Domain	Key findings	Clinical implications
Prevalence	Depression affects 20–37% of PAD patients; pooled ~24–25% in meta-analyses (Abi-Jaoudé et al., 2022 [12]; Scierka et al., 2023 [13]; Shakt et al., 2024 [14])	Routine depression screening in PAD clinics
Mortality and amputation	Depression ↑ mortality 25–70%, ~2× amputation risk (Arya et al., 2018 [15]; Scierka et al., 2023 [13]; Shakt et al., 2024 [14])	Integrate psychological care to improve survival and limb outcomes
Functional outcomes	Depression → reduced walking distance, poorer post-revascularization recovery (McDermott et al., 2016 [9]; Cherr et al, 2007 [4]; Smolderen et al., 2011 [20]; Welch et al., 2023 [11])	Consider psychological status in revascularization and rehabilitation planning
Mechanisms	Behavioral (non-adherence, smoking, inactivity) + Biological (HPA axis, inflammation, endothelial dysfunction) + Genetic (via BMI, smoking) (Jia et al., 2019 [16]; Brostow et al., 2017 [21]; Smolderen et al., 2023 [5])	Target interventions addressing both biological and behavioral risks
Antidepressant therapy	Treated depression shows lower risk than untreated but outcomes remain worse than non-depressed (Arya et al., 2018 [15]; Golledge et al., 2025 [22]; Brostow et al., 2017 [21])	Multimodal care (pharmacotherapy + exercise + CBT) likely most effective

## Conclusions

The high prevalence and significant impact of depression on PAD outcomes emphasize the need for routine screening, particularly in patients with severe disease or those undergoing revascularization procedures. Brief, validated instruments such as the PHQ-2 or PHQ-9 are recommended for this purpose, as they are practical for use in vascular clinics and supported by cardiovascular guidelines. Early identification of depressive symptoms can enable timely intervention and improve both psychological and physical outcomes. Furthermore, integrating depression management into PAD care may enhance treatment adherence, functional status, and potentially reduce mortality and amputation rates. Nevertheless, further research is warranted to better elucidate the role of depression screening and management in this specific patient population.
